# Quantified MRI and 25OH-VitD3 can be used as effective biomarkers for patients with neoadjuvant chemotherapy-induced liver injury in CRCLM?

**DOI:** 10.1186/s12885-020-07282-6

**Published:** 2020-08-15

**Authors:** Qian Wang, Feng Ye, Peiqing Ma, Yiqun Che, Weilan Guo, Dong Yan, Xinming Zhao

**Affiliations:** 1 Department of imaging diagnosis, National Cancer Center/National Clinical Research Center for Cancer/Cancer Hospital, Chinese Academy of Medical Sciences, Beijing, China; 2 Department of Pathology, National Cancer Center/National Clinical Research Center for Cancer/Cancer Hospital, Chinese Academy of Medical Sciences, Beijing, China; 3 Department of Clinical Laboratory, National Cancer Center/National Clinical Research Center for Cancer/Cancer Hospital, Chinese Academy of Medical Sciences, Beijing, China; 4 Department of Epidemiology, National Cancer Center/National Clinical Research Center for Cancer/Cancer Hospital, Chinese Academy of Medical Sciences, Beijing, China; 5 Department of Interventional Therapy, National Cancer Center/National Clinical Research Center for Cancer/Cancer Hospital, Chinese Academy of Medical Sciences, Beijing, China

**Keywords:** CRCLM, Liver injury, Steatosis, SOS, Biomarkers, MRI, VitminD

## Abstract

**Background:**

To evaluate proton-density fat-fraction (PDFF) and intravoxel incoherent motion (IVIM) techniques, and human 25-hydroxyvitamin D3 (25OH-VitD3) levels, as potential biomarkers in patients with colorectal cancer with liver metastasis (CRCLM). Changes were compared with those related to chemotherapy-associated steatohepatitis (CASH) and sinusoidal obstruction syndrome (SOS).

**Methods:**

63 patients with pathologically confirmed colorectal adenocarcinoma received 4–6 courses of NC before liver resection and underwent magnetic resonance imaging (MRI) with iterative decomposition of water and fat with echo asymmetry and least-squares estimation quantification and IVIM sequences. Blood samples were analyzed using CTCAE. Pathological changes of liver tissues outside the metastases were assessed as the gold standard, and receiver operating characteristic (ROC) curves were analyzed.

**Results:**

16 cases had CASH liver injury, 14 cases had SOS changes, and 4 cases had CASH and SOS, and 7 showed no significant changes. Consistency between biochemical indices and pathological findings was poor (*kappa* = 0.246, *p* = 0.005). The areas under the ROC curve (AUCs) of ALT, AST, ALP, GGT, and TBIL were 0.571–0.691. AUCs of D, FF, and 25OH-VitD3 exceeded 0.8; when considering these markers together, sensitivity was 85.29% and specificity was 93.13%. ANOVA showed statistically significant differences among *D*, FF, and 25OH-VitD3 for different grades of liver injury (*F* = 4.64–26.5, *p* = 0.000–0.016).

**Conclusions:**

*D*, FF, and 25OH-VitD3 are biomarkers for accurate prediction of NC-induced liver injury in patients with CRCLM, while FF and 25OH-VitD3 might be beneficial to distinguish liver injury grades.

**Trial registration:**

Current Trials was retrospectively registered as ChiCTR1800015242 at Chinese Clinical Trial Registry on March 16, 2018.

## Background

Colorectal cancer (CRC) is a leading cause of cancer-related mortality in the world, with over 1.2 million new cases diagnosed each year [1]. Approximately 50% of these patients develop involvement of the liver during the disease course and some with colorectal liver-only metastases may undergo liver metastasectomy [2, 3].

CLM treatment has advanced considerably, both in terms of surgical resection and neoteric chemotherapeutic regimes [4–6]. Neoadjuvant chemotherapy (NC) provides an opportunity for cure with hepatic resection in selected patients with CLM. Extensive use of multiple chemotherapeutic agents has resulted in consensus regarding distinct hepatotoxicity patterns, including CASH and SOS, which are associated with specific drugs [7–9]. 5-Fluorouracil (5-FU) is a fluoropyrimidine antimetabolite, and it induces impaired-oxidation and accumulation of fatty acids and causes hepatic steatosis [10, 11]. Irinotecan is a camptothecin analogue, which mainly leads to mitochondrial impairment and inflammation secondary to cytokine release; it causes steatohepatitis [10, 12]. Oxaliplatin is a platinum-based compound that generates reactive oxygen species (ROS) and causes glutathione depletion, causing SOS. The related antibody-drug, including cetuximab and bevacizumab, were reported to cause no recognized hepatotoxicity in recent clinical trials [10, 13–15].

Liver resectability after NC depends not only on anatomic and oncological factors, but also on a liver remnant with sufficient volume and adequate blood perfusion and biliary drainage, according to the degree of histopathological NC-induced liver injury [16–20]. NC-induced liver injury is routinely diagnosed according to serum alanine aminotransferase (ALT) and total bilirubin (TBIL) levels [21]. Currently, contrast-enhanced CT and MRI are recommended as more advantageous qualitative approaches [22–25]. However, the clinical presentation of NC-induced liver injury requires more intuitive, specific, and quantitative biomarkers for a precise preoperative assessment of liver injury.

To date, there are some reports describing quantitative indexes of imaging biomarkers and specific serum biomarkers in non-alcoholic fatty liver disease (NAFLD) or alcoholic fatty liver disease (AFLD). However, these imaging and special serum biomarkers have not been reported to evaluate chemotherapy-related liver injury. Thus, the purpose of this study was to describe the value of MRI biomarkers and human 25OH-VitD3 as quantitative biomarkers to evaluate liver injury in patients with CLM who underwent NC before liver resection.

## Methods

### Patient population

**Fig. 1:**
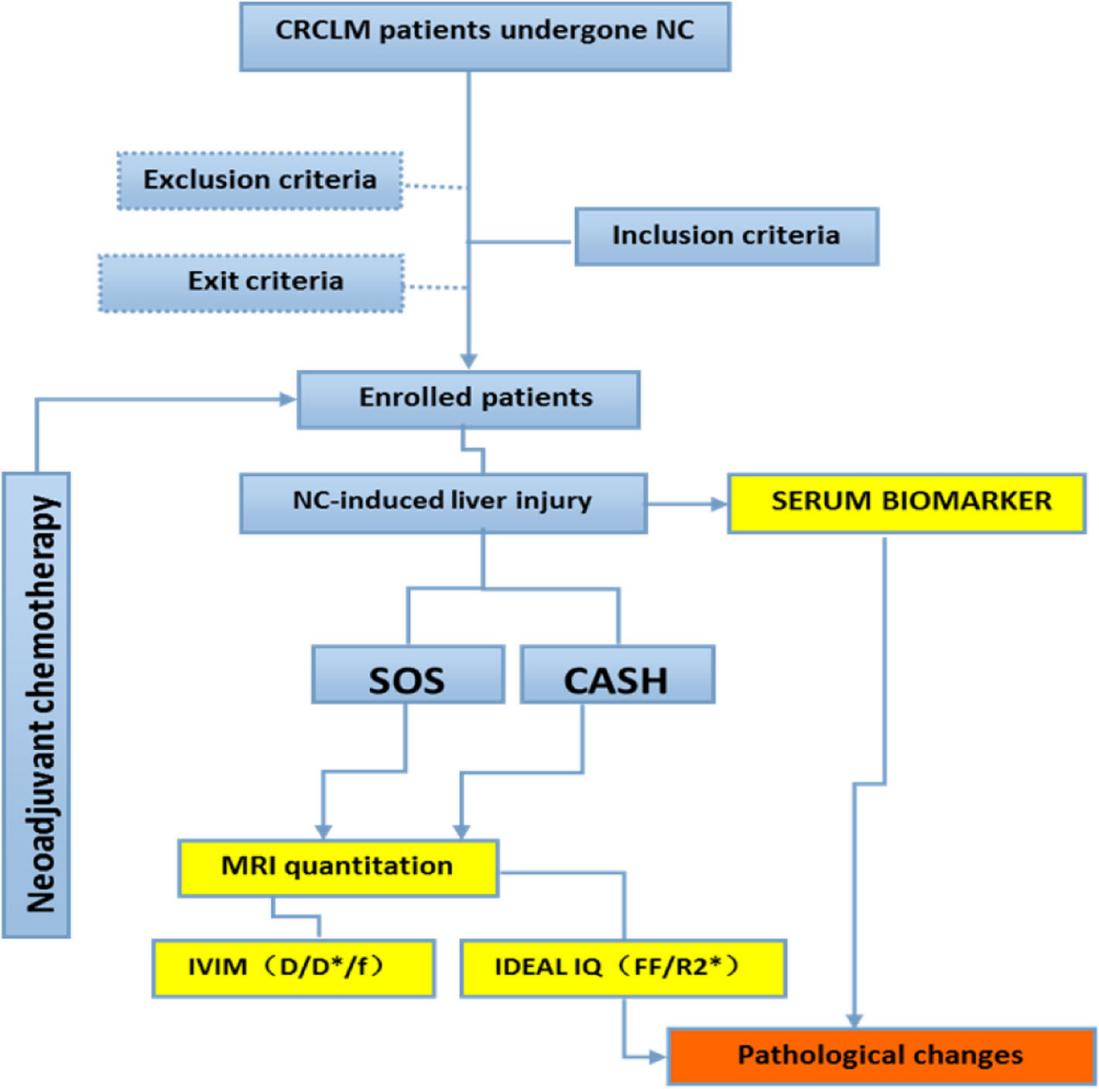
Chart of study enrollment and study flow

Figure 1 showed the patient selection process, who were enrolled in our hospital between August 2015 and July 2016. The single-center study was approved by China cancer foundation ethics committee (NCC2016YQ-17). Patients who enrolled in this study were gave written informed consent and volunteered to participate. And inclusion and exclusion criteria were described in Supplementary Materials. All patients underwent MRI-IVIM and MRI-PDFF examinations to determine the baseline liver state before NC and pre-surgery. NC regimen details were shown in the Supplementary Materials.

### Serum collection

In all patients, ALT, aspartate transaminase (AST), alkaline phosphatase (ALP), gamma-glutamyl transpeptidase (GGT), and TBIL levels in serum were measured. Blood samples of 25OH-VitD3 measurement were obtained after a 12-h fast by venipuncture of the large antecubital veins, without stasis, before NC and pre-operation. The samples were centrifuged immediately, and the plasma separated and stored at − 80 °C, and all samples were studied on the same day using the ELISA kit (25OH Vitamin D Total ELISA, Britain immunodiagnostic systems limited. No.AC-57F1, sensitivity 5 nmol/L, within-run precision *CV* 5.3–6.7%, Batch precision *CV* 4.6–8.7%). The standard institutional reference ranges for blood sample parameters were used. The previously described serum diagnostic criteria of CTCAE recommended by the Council for International Organizations of Medical Sciences (CIOMS) were used to assess liver function. 25OH-VitD3 levels were evaluated using a 3-point scale [26]: (1 = deficient, concentration < 10 ng/ml; 2 = insufficient, 10 ng/ml < concentration < 29 ng/ml; 3 = sufficient, 30 ng/ml < concentration < 100 ng/ml).

### MRI collection

Baseline and post-chemotherapy MRI was performed to assess CASH and SOS; PDFF-MRI and IVIM-MRI technique were used. All patients were examined with a 3.0-T MR system (Discovery MR 750; GE, Milwaukee, WI, USA) using 8-channel phased-array coils with respiratory gating. A multi-echo 3D SPGR sequence with fly-back gradients were employed (IDEAL IQ, GE) to evaluate CASH. The IVIM imaging sequence was based on a single-shot DW spin-echo-type echo-planar imaging sequence for the evaluation of SOS. The detail protocol was in Supplementary Materials.

All fat-fraction maps were used to estimate the hepatic fat-fraction, and the signal intensity from regions-of-interest (ROI) in the liver was calculated by two radiologists (Y.D. and F.Y. together, 10 years’ experience with diagnostic imaging). A 1-cm^2^ ROI was placed in the liver parenchyma around the metastatic tumor, avoiding bile ducts,blood vessels and chemotherapy response zone [27]. All IVIM images were transferred to a workstation (View 10.0, GE). Two radiologists (L.G. and F.Y., 10 years’ experience with diagnostic imaging) drew ROIs of approximately 300–400 mm^2^ at similar sites in fat-fraction maps [28, 29]. All ROIs were manually positioned on the b = 0 DW images. *D* is the “true” diffusion coefficient, representing pure molecular diffusion, and D* is the “pseudo” diffusion coefficient, representing incoherent microcirculation within the voxel, *f* is the perfusion fraction of the pseudo-diffusion linked to microcirculation in the ROI [30–32].

### Pathology collection

Sufficient extra tissues outside liver metastasis were performed for CASH and SOS assessment by a faculty hepatopathologist, who was blinded to clinical and radiological data (PQ.M., with 10 years of experience), scored the severe degree CASH and SOS. The mean of the two near-continuous CASH and SOS scores were recorded and converted to a 4-point scale [33, 34]. Severity of CASH was evaluated according to the degree of hepatocyte steatosis (4-point scale: 0 = none, no hepatic steatosis; 1 = mild, < 33% of the total area was involved; 2 = moderate, 33–67% was involved; and 3 = severe, > 67% was involved). Severity of SOS was evaluated according to the degree of sinusoidal obstruction (4-point scale: 0 = none, no sinusoidal obstruction; 1 = mild, < one-third sinusoidal obstruction in the affected center lobule; 3 = moderate, < two-thirds was involved; and 4 = severe, complete sinusoidal obstruction).

### Statistical analysis

Patient samples were estimated using a two-sided Z test by PASS15.0 software. Rank variables were as numbers and percentages, continuous variables as means±SD. Kruskal-wallis test was used to compare the differences between groups for data that did not meet the normal modality. ROC analysis was used to determine the discriminatory capability. Inter-observer agreement was determined by Cohen’s kappa coefficient. *P* < 0.05 was considered statistically significant. Statistical analysis was performed by a biostatistical analyst (LW.G., with 5 years of experience) using R software version 3.3.2.

## Results

### Patients

63 subjects diagnosed with CRCLM were included. Cohort characteristics are summarized in Table 1 and described in the Supplementary Materials. Of the 63 patients, 41 subjects with histopathological samples were analyzed further; these included 22 men (54%) and 19 women (46%), with a mean age of 54.4 years (range 32–71 years). Mean body mass index was 27.8 kg/m^2^ (range, 18.4–33.1 kg/m^2^). Blood specimens and MR images were obtained within 3 days before NC and before liver resection (or RFA), respectively. NC regimens were as follows: FOLFOX (*n* = 24), FOLFOX and cetuximab (*n* = 4), FOLFIRI (*n* = 17), FOLFIRI and bevacizumab (n = 4). The NC duration ranged from 42 to 130 days (median, 86 days).

**Table 1:** Patient Characteristics

Characteristic	Value
General information	
Patient sex	
Male	22 (53.7%)
Female	19 (46.3%)
Mean age(y)*	54.4 ± 10.2 (32.0, 71.0)
BMI (kg/m^2^)*	27.8 ± 4.3 (18.4, 33.1)
**NC regimen**	
FOLFOX	20 (48.7%)
FOLFOX and cetuximab	4 (9.8%)
FOLFIRI	13 (31.7%)
FOLFIRI and bevacizumab	4 (9.8%)
**CASH scores**	
Grade 0	8 (19.5%)
Grade 1	22 (53.7%)
Grade 2	11 (26.8%)
Grade 3	–
**CASH scores**	
Grade 0	10 (24.4%)
Grade 1	23 (56.1%)
Grade 2	8 (9.76%)
Grade 3	–
**CASH & SOS scores**	
Grade 0	7 (17.1%)
Grade 1	23 (56.1%)
Grade 2	11 (26.8%)
Grade 3	

*Data are given as number (percentage) or mean ± standard deviation, with ranges in parentheses

### Pathological assessment

Liver sections were stained with hematoxylin and eosin. The maximum cross-sectional area of tissue strips in 11 RFA cases ranged from 0.4 × 1.0 cm to 2.2 × 1.0 cm. Microscopicaly, at least three portal areas were observed, meeting with diagnostic requirements. Histopathological features are shown in Table 2A.

**Table 2:** Histopathological changes of patients with CRCLM after NC

**2A Histopathological features**			**Value**	**Percentage (%)**
Hepatocellular ballooning			31	75.6
Focal or bridging necrosis			28	68.9
Eosinophilic infiltration			29	71.1
Bile ducts damage			22	53.3
Hepatic sinus widening			15	37.8
Kupper hyperplasia			10	24.4
Collagen accumulation			2	4.44
Clastic necrosis in the portal area			2	4.44
**2B His-Scores**	**Grade 0**	**Grade 1**	**Grade2**	**Grade 3**
CASH*	8 (19.5%)	22 (53.7%)	11 (26.8%)	–
SOS*	10 (24.4%)	23 (56.1%)	8 (9.76%)	–
CASH & SOS	7 (17.1%)	23 (56.1%)	11 (26.8%)	–

*CASH, nonalcoholic steatohepatitis, *SOS* sinusoidal obstruction syndrome

And 41 patients underwent pathology grading; these details are shown in Table 2B. Almost half of the patients were CASH grade 1, SOS grade 1, and CASH & SOS grade 1. The combined score was used as the criterion for NC-induced liver injury; 34 patients were diagnosed with mild to moderate liver injury.

### MRI/serum biomarker assessment

Blood biochemical results of the 41 patients were evaluated according to the CTCAE as recommended by the CIOMS for liver injury. Nineteen cases were diagnosed with abnormal liver function. Of 19 patients, 13 cases were diagnosed as having the hepatocellular-type and 6 cases were diagnosed as having the cholestasis-type, with 22 patients being grade 0; 11 patients being grade 1; 7 patients being grade 2, and 1 patient being grade 3 (Table 3A). Blood biochemical results and histological outcome scores were analyzed for consistency; the *Kappa* value was 0.246 (*p* = 0.005), indicating poor consistency. The blood biochemical index significantly underestimated the histopathological changes of liver injury (Table 3B).

**Table 3:** Serum assessments of patients with CRCLM after NC by CTCAE

**3A**	**Liver function scales**				
**Blood biochemical index**	**Abnormal liver function**				
	**Grade 0**	**Grade 1**	**Grade 2**		**Grade 3**
ALT*	–	11	5		1
AST*	–	10	3		1
ALP*	–	7	2		1
TBIL*	–	6	3		1
**Total**	22	11	7		1
**3B**	**His-scores liver injury**				**Total**
**NCI.CTC scores**	**Grade 0**	**Grade 1**	**Grade 2**	**Grade 3**	
**Grade 0**	6	13	3	–	22
**Grade 1**	1	8	2	–	11
**Grade 2**	–	2	5	–	7
**Grade 3**	–	–	1	–	1
**Total**	7	23	11	–	41

*alanine aminotransferase (ALT), grass transaminase (AST), alkaline phosphatase (ALP), gamma-glutamyl transpeptidase (GGT), total bilirubin (TBIL)

ROC analysis was performed for related blood biochemical indexes, including ALT, AST, ALP, GGT, and TBIL, to differentiate dichotomously histological outcomes. The AUCs were as follows: ALT, 0.685 (95% CI: 0.511, 0.858); AST, 0.691 (95% CI: 0.496, 0.886); ALP, 0.574 (95% CI: 0.335, 0.812); GGT, 0.571 (95% CI: 0.367, 0.780), and TBIL, 0.626 (95% CI: 0.398, 0.854).

25OH-VitD3 among these patients ranged from 3.4 ng/ml to 14.5 ng/ml. 25OH-VitD3 had an AUC of 0.868 (95% CI: 0.736, 0.999) (Table 4**)**. There was a significant difference before and after NC (*p* < 0.01).

**Table 4:** ROC analysis of blood biochemical indexes, MRI-PDFF, MRI-IVIM and 25OH-VitD3 diagnosing NC-induced liver injury

	AUC	95% CI		***P***	Std Error
ALT	0.685	0.511	0.858	0.127	0.089
**AST**	0.691	0.496	0.886	0.115	0.099
**ALP**	0.574	0.335	0.812	0.544	0.122
**GGT**	0.571	0.367	0.780	0.541	0.105
**TBIL**	0.626	0.398	0.854	0.299	0.116
**25OH-VitD3**	0.868	0.736	0.999	0.002*	0.067
***D***	0.824	0.663	0.984	0.008*	0.082
D*	0.605	0.355	0.855	0.386	0.128
***f***	0.501	0.315	0.685	0.997	0.094
**FF**	0.962	0.899	1.000	0.000*	0.032

* is from IVIM-MRI technique, and meaning perfusion diffusion coefficient

The MRI fat-fraction (FF) values among these patients before and after NC were 4.41 ± 1.456 and 12.147 ± 5.272, respectively, and FF had an AUC of 0.962 (95% CI: 0.899, 1.000). The trend for increasing FF was statistically significant (*p* < 0.01).

**Fig. 2:**
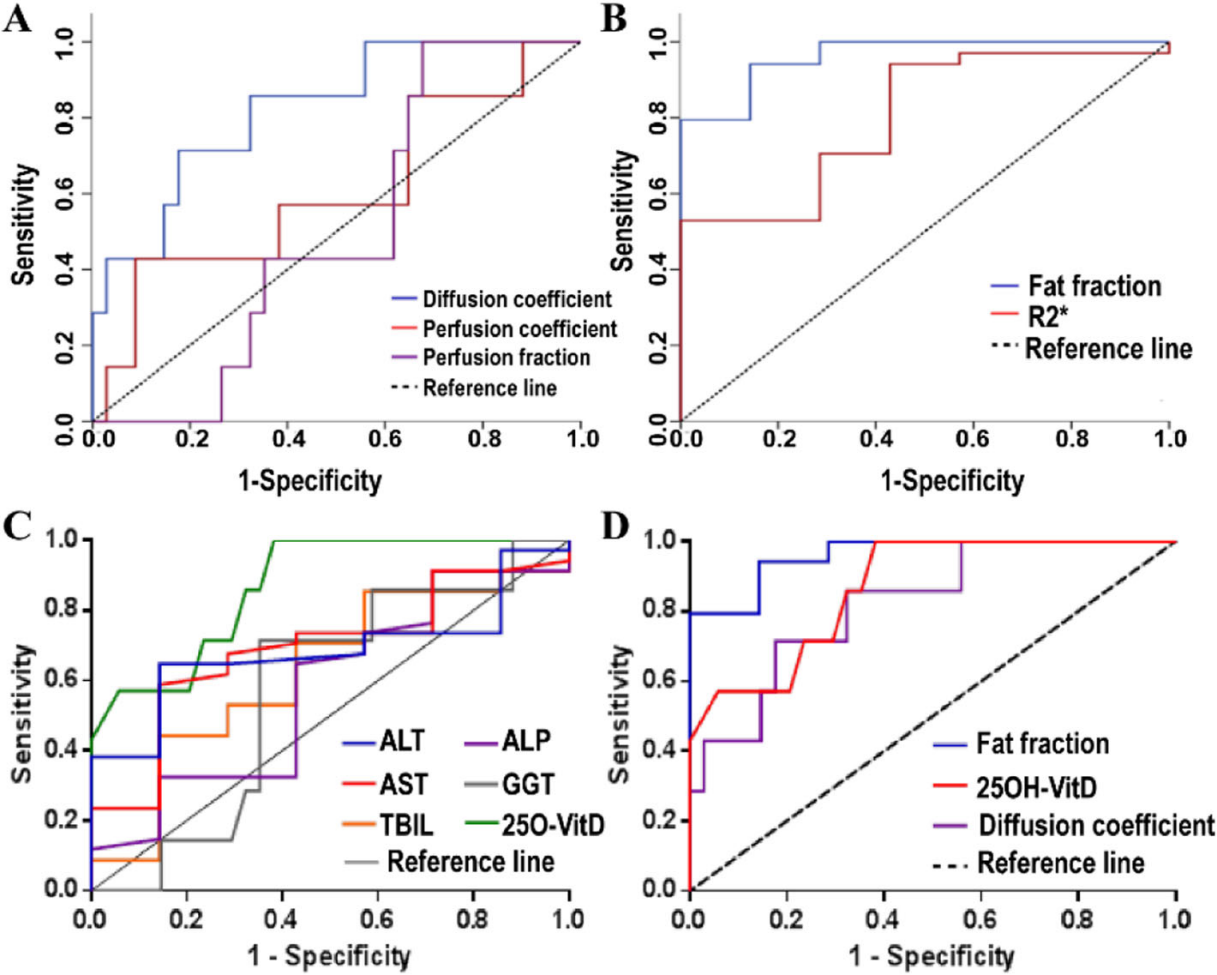
The ROC curve demonstrates that diffusion coefficient has a high accuracy among IVIM-DWI imaging markers, with an AUC of 0.824 as shown in **a**, fat fraction has a high accuracy by PDFF technique, with an AUC of 0.962 in **b**, 25OH-VitD has a high accuracy among the blood indexes with an AUC of 0.868 as shown in **c**, and the AUC curves of the best biomarkers among these imaging and blood indexes are shown in **d**

IVIM imaging data were acquired for all subjects. No data in this study suffered from severe motion-induced displacements or artifacts. The *D*, D*, and *f* (× 10^− 3^ mm^2^/s) were measured. The *D* values before and after NC were 1.201 ± 0.362 and 0.771 ± 0.195, respectively, and *D* had an AUC of 0.824 (95% CI: 0.633, 0.984). The decreasing trend for *D* was statistically significant (*p* < 0.01). The D* values before and after NC were 10.167 ± 2.024 and 8.605 ± 0.973, respectively, and D* had an AUC of 0.625 (95% CI: 0.355, 0.855). The *f* values before and after NC were 0.144 ± 0.021 and 0.141 ± 0.016, and *f* had an AUC of 0.501 (95% CI: 0.315, 0.685). The changes in D* and *f* before and after NC were not statistically significantly different (*p* >  0.01) (Table 4**)**. Thus, 25OH-VitD3, FF, and *D* could better predict the pathological outcomes in CRCLM patients who underwent NC. The AUC curves of the best biomarkers among these imaging and blood indexes are shown in Fig. 2.

### MRI/serum diagnostic threshold for NC-induced liver injury

Pathological grading of liver injury (grades 0–2) was used as the regional standard and the reported means [33, 34]. 25OH-VitD3, *D*, and FF values are statistically described in Table S1. Comparison among patients with NC-induced liver injury (grades 0–2) showed statistically significant differences for 25OH-VitD3, *D*, and FF values, with *F* values of 4.642 to 26.050, and *p* < 0.05. Analysis of variance for comparison of the three effective biomarkers among groups of liver injury grades were shown in Table 5A.

**Table 5:** ANOVA analysis s and Threshold intervals for NC-induced liver injury

**5A**	**Effective biomarker**	**Block**	**Sum of Squares**	**df**	**Mean Square**	***F*** **value**	***P*** **value**
**25OH-VitD3**		**Between Groups**	153.670	2	76.835	26.050	.000*
		**Within Groups**	112.080	38	2.949		
		**Total**	265.750	40			
**FF**		**Between Groups**	719.563	2	359.782	34.830	.000*
		**Within Groups**	392.531	38	10.330		
		**Total**	1112.094	40			
***D***		**Between Groups**	.299	2	.150	4.642	.016*
		**Within Groups**	1.225	38	.032		
		**Total**	1.524	40			
**5B**	**Effective biomarker**		**NC-induced liver injury**				
			**Grade 0**		**Grade 1**	**Grade 2**	
**25OH-VitD3**			**>** 10.65		> 6.65; ≤ 10.65	≤ 6.65	
**FF**			≤ 7.47		> 7.47; ≤ 15.84	**>** 15.84	
***D***			**>** 0.985		> 0.674; ≤ 0.985	≤ 0.674	

* is from IVIM-MRI technique, and meaning perfusion diffusion coefficient

**Fig. 3:**
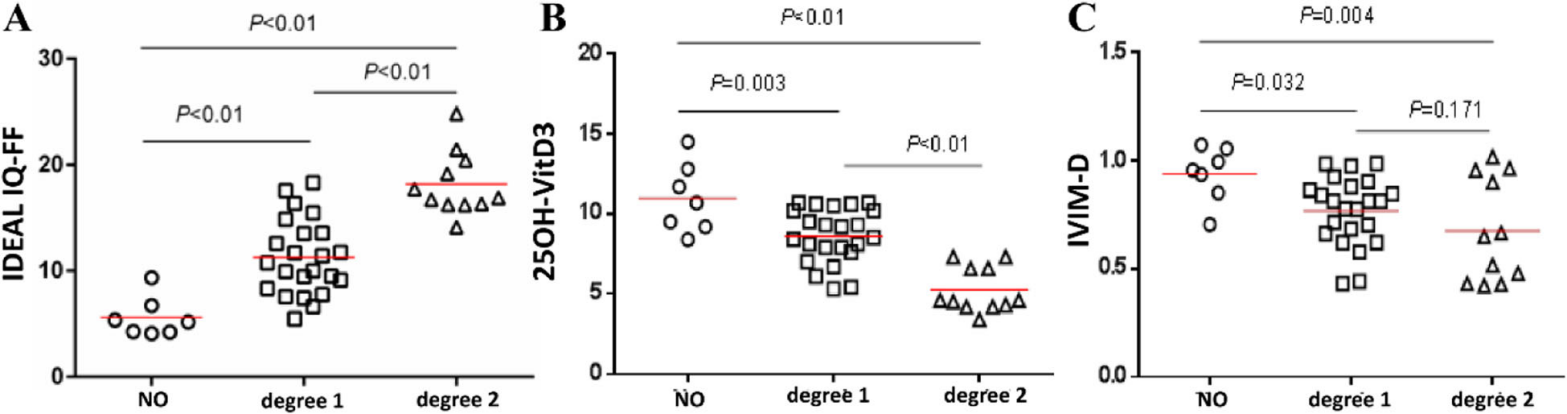
Effective biomarkers distributed in different NC-induced liver injury degrees (Degree 0–2). **a**, IDEAL-IQ FF values in NC-induced liver injury degrees and there is statistically significant difference among three degrees (*p* < 0.01), **b**, 25OH-VitD3 values in NC-induced liver injury degrees and there is statistically significant difference among three degrees (*p* < 0.01), **c**, IVIM-D values in NC-induced liver injury degrees and there is statistically significant difference among three degrees (*p* = 0.004). However, there is no statistically significant difference between degree 1 and 2 (*p* = 0.17)

Distribution of effective biomarkers in NC-induced liver injury was shown in Fig. 3 and Table 5B; 83% of subjects (34/41) were classified as having the same NC-induced liver injury when using the effective biomarker-derived thresholds. There were statistically significant differences among the different degrees of NC-induced liver injury (degree 0–2) for FF (*p* < 0.01) and 25OH-VitD3 (*p* < 0.01). However, IVIM-D could not distinguish between NC-induced liver injury of grades 1 and 2 (*p* = 0.171).

Tandem diagnosis test of combining 25OH-VitD3, D*,* and FF for with or without NC-induced liver injury provided 85.29% sensitivity and 93.13% specificity.

## Discussion

Our study demonstrates that partial IVIM-DWI biomarkers, like D, FF, and 25OH-VitD3 are linked to the histological change with NC-induced liver injury, and correlates with the degrees of NC-induced liver injury in patients with CRCLM. Perfusion is decreased in liver after NC and be related to hepatic SOS. Fat content in liver is meanwhile increased after NC and is perhaps concerned with hepatic steatosis. As liver injury with NC increases, the levels of 25OH-VitD3 continues to fall. These features might therefore be good biomarkers for the severity of liver injury with NC.

Liver injury is a hallmark of chemotherapy side effects in normal liver tissues outside tumor and a major contributor to the complications and mortality after resection for liver metastasis in CRCLM patients [2, 4]. Currently, liver injury after NC is considered as the congestion of hepatic sinus syndrome and hepatic steatosis occur simultaneously [12, 33, 34]. Hepatic steatosis can be used as an independent predictor of postoperative complications. Other studies have shown that moderate or more severe SOS will increase the risk of postoperative complications, mainly infection and bleeding [35, 36]. Therefore, it is important to determine the timing, mode, and prognosis of surgery by observing the state of the liver as a whole, using intuitive imaging methods, performing a non-invasive quantitative evaluation before surgery [37].

Available biomarkers for detecting liver injury with NC and accurately grading liver injury might allow for new markers and liver injury prediction. Traditional tools and conventional imaging are still incomplete and insufficient for detecting liver injury with NC. Blood test enables liver quality in a certain degree. However, these indexes can’t directly reflect the liver damage caused by chemotherapy and might be disturbed by some physiological or pathological states. The utility of CT or MR imaging for liver injury is limited by the observer-dependent. And the efficiency of some imaging biomarkers like CT value only reflect the part, not the whole. Although several novel imaging approaches have been studied, no single modality, currently, can roundly and accurately assess and grade liver injury with NC [35–37].

Pathologically, chemotherapy side effect with the excessive deposition of collagen fibers in the extracellular matrix and the proliferation of fibrous tissue, slight dilatation and congestion in the hepatic sinus around the portal area. Meanwhile, hepatocytes around the central vein are loose and swollen, with focal steatosis and punctate necrosis. These pathological changes in our study are consistent with the reported expert consensus [23], and might cause a reduction in effective blood perfusion and an increase in fat content within liver parenchyma [24]. Previous studies using IVIM-DWI demonstrated the change of effective blood volume before and after chemotherapy and liver fibrosis [14, 17], and using PDFF and 25OH-VitD3 demonstrated an increase in fat content of patients with NAFLD [33–35, 38]. Hence, the quantification of blood perfusion and fat content within liver parenchyma might help to diagnose and grade liver damage related NC in patients with CRCLM.

**Fig. 4:**
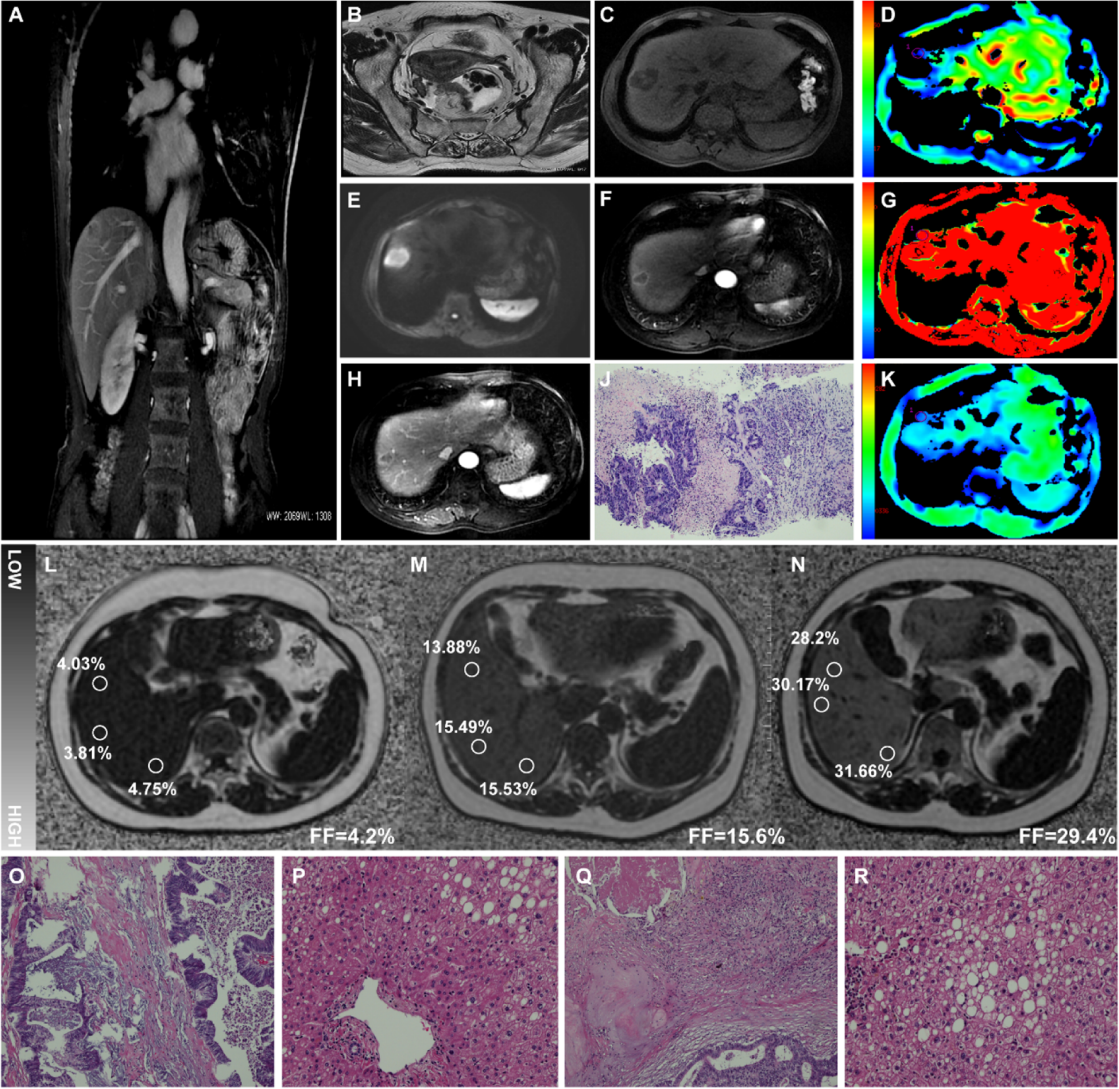
A 56-year-old woman with CRCLM, **a**, a coronal contrast-weighted image and **b**, an Axial T2-weighted image showed that the doubtful neoplasm near the rectum infiltrates the surrounding fat space. The doubtful neoplasm could be distinguished in T1-weighted and contrast-weighted images were shown in **c**, and **f**, **h**. And the DWI and IVIM-DWI images were shown and measured in **d**, **g** and **k**. Subsequently, liver biopsy for the doubtful neoplasm was performed and a small number of histological biopsy samples confirmed liver tumor as rectal adenocarcinoma liver metastasis. MRI imaging PDFF maps and NC-induced histopathological changes in a patient, 48-year-old man, who was diagnosed as CRCLM and underwent 6 courses of NC before liver metastatic tumor resection. **l**, the baseline map of fat fraction before NC, **m**, the FF map after 2 courses of NC, **n**, the FF map before liver resection within 3 days. H&E staining sections of liver metastatic tumor and hepatic parenchyma extra tumor in liver. **o**, Chemotherapeutic reactivity is characterized by a large amount of clastic necrosis in the liver tissue outside the tumor, with lymphocyte and plasma cell infiltration, **p**, Small bile duct hyperplasia with or without mild dilation is seen in the portal area, **q**, Tumor degenerative necrosis caused by chemotherapeutic reaction, with fibrous tissue hyperplasia and hyaline degeneration, collagen accumulation and inflammatory cell infiltration, **r**, Slight dilatation and congestion in the hepatic sinus around the portal area, steatohepatitis and large lipid droplets are deposited

IVIM-DWI is a non-invasive MRI technique which can provide quantitative information about blood perfusion via fractional perfusion and perfusion coefficient without contrast administration [39]. In IVIM characterizing chemotherapy evaluation for hepatocellular carcinoma, previous investigators found a significant reduce in the diffusion coefficient of post-therapy compared with that of pre-therapy in the effective group [40]. And the assessments of fractional perfusion and perfusion coefficient weren’t stable [41]. Other studies showed that significantly lower fractional perfusion, higher perfusion coefficient in diffuse liver fibrosis. And Fractional perfusion has been shown to decrease with increasing liver fibrosis stages. Our study demonstrated a slightly decrease of diffusion coefficient in liver parenchyma before and after NC, and no significant difference in fractional perfusion and perfusion coefficient in liver parenchyma before and after NC in Fig. 4. These might be explained by the reason that liver damage causes lobular inflammation, deposition of collagen fibers, and hepatic sinus obstruction, which hamper the Brownian motion of the water molecule and thus infect diffusion coefficient [42]. Moreover, diffusion coefficient was only accurate for differentiating mild-moderate liver damage from no liver injury. That is to say, it can diagnosed but can’t grade accurately the different liver injury with NC. This may be due to the patients in our study are given short-term preoperative chemotherapy.

MRI-PDFF is proved to be a highly sensitive and specific predictor of the quantification of fat content and many studies have shown the application of this technique in early detection of NAFLD [26]. So, PDFF may be a potential aid in liver injury in the patients with CRCLM, based on similar pathological changes. As liver damage worsens, it is shown that the levels of fat content gradually increased in our study, from 4.41% up to 12.15%. Importantly, the cutoff threshold for NC-induced liver injury in MRI-PDFF was 7.47% in our study, which is higher than 5.6% to define hepatic steatosis in adults [27]. Data from our study suggest that fat deposition in hepatocyte increases gradually as chemotherapy duration accumulates in Fig. 4. More importantly, PDFF can accurately distinguish mild, moderate from no NC-induced liver injury. This observation not only strengthens previous findings that PDFF can accurate quantify liver fat content, but also reveals that fat quantification by PDFF can effectively monitor hepatic steatosis associated with NC-induced liver injury in patients with CRCLM.

Interestingly, Levels of ALT is usually a marker of hepatocellular injury [43]. However, levels of blood biochemical indexes, like ALT, AST, ALP, GGT, and TBIL, are not able to predict NC-induced liver injury in our study. This might be connected with mild-moderate liver injury in some patients who are given short-term NC treatment. ALT is limited as a predictor of hepatic steatosis in this population. This observation for ALT suggests that increasing liver fat content may not induce serious hepatocellular injury in these subjects. And while ALT may be a useful marker of hepatocellular injury once NC-induced liver injury has been identified. Currently, it may be unscientific to characterize NC-induced liver injury in patients with CRCLM only by blood biochemical indexes. 25OH-VitD3 is the best indicator of vitamin D reserves, as it has no biological activity and has a long and stable half-life in the blood [38]. 25OH-VitD3 was associated with the occurrence and severity of NAFLD as shown in few studies [44]. 25OH-VitD3 is a sensitive prediction for NC-induced liver injury. And the levels of 25OH-VitD3 in these population are lower than 30 ng/ml to define normality in adults [38]. 25OH-VitD3 can accurately distinguish mild, moderate from no NC-induced liver injury in our study.

A unique contribution of this study is the simultaneous acquisition of both imaging and serum markers in the patients with CRCLM. The changes of liver parenchyma after NC, such as NASH and SOS, are very complicated and should be given attention to, which is closely related with intraoperative risk and postoperative complications about hepatectomy. The comprehensive clinical risk assessment before liver surgery with diagnosis imaging and sensitive blood index in the quantitative and intuitional evaluation of liver statue may allow for more accurate detection than current clinical application.

A limitation of this study is smaller sample size because of presentable and incomplete clinical data. Otherwise, pathological evaluation was also limited to normal liver tissue around the metastatic tumor. However, the pathological sections of liver metastases after neo-adjuvant chemotherapy were observed by pathology expert involved in our study. The number of hepatocytes and the proportion of extracellular matrix in non-neoplastic liver tissues outside the chemotherapy response zone fully satisfied our microscopic pathological evaluation.

## Conclusion

In this study, diffusion coefficient *D*, fat fraction FF, and 25OH-VitD3 were in accurately diagnosing and grading NC-induced liver injury in the patients with CRCLM, which might be potential biomarkers in patients who NC-induced liver injury with CRCLM, beneficial to hepatic operation opportunity and the risk prediction of post-operation.

## Supplementary information

**Additional file 1. **

## Data Availability

The datasets generated and analysed during the current study are not publicly available as these contain individual person’s data but are available from the corresponding author on reasonable request, after pseudonymization of the data and legal agreement.

## Abbreviations

25OH-VitD3: 25-HydroxyvitaminD3
AUC: area under the ROC curve
CASH: chemotherapy-associated steatohepatitis
CI: confidence interval
CRCLM: colorectal cancer with liver metastasis
CTCAE: the Common Terminology Criteria for Adverse Events
IDEAL IQ: Iterative decomposition of water and fat with echo asymmetry and least-squares estimation quantification
IVIM: Intravoxel incoherent motion
NC: neoadjuvant chemotherapy
NPV: negative predictive value
PDFF: proton-density fat-fraction
PPV: positive-predictive value
ROC: receiver operating characteristic
SOS: sinusoidal obstruction syndrome
