# Supplementary material for: Quantified MRI and 25OH-VitD3 can be used as effective biomarkers for patients with neoadjuvant chemotherapy-induced liver injury in CRCLM?
Source: BMC Cancer. 2020 Aug 15;20:767. doi: 10.1186/s12885-020-07282-6 (PMC7429712; doi:10.1186/s12885-020-07282-6)
Supplement: Supplementary file 1 — Additional file 1. [file 12885_2020_7282_MOESM1_ESM.docx]

**Supplementary materials**

**Quantified MRI and 25OH-VitD3 can be used as effective biomarkers for patients with neoadjuvant chemotherapy-induced liver injury in CRCLM?**

**Materials and methods**

**Inclusion and Exclusion Criteria**

Inclusion criteria: Age 18 years or older; advanced colorectal cancer with hepatic metastasis (TXNXM1) highly suspected by cytology, histopathology or combined radiology; laboratory examination were performed for heart, liver, kidney and bone marrow functions were basically normal; receive at least 4 to 6 cycles of combination chemotherapy; liver ultrasound examination were normal before study.

Exclusion criteria: Subjects were excluded if they had evidence of the forms of drug-induced liver injury; hematopathy and a history of repeated massive blood transfusion; not very well tolerated MRI examinations. And few subjects were deciduous from the study because unwilling to continue or giving up treatment halfway.

**Neoadjuvant Chemotherapy Regimes**

The NC regimens of the 63 patients were as follows: fluorouracil (FU) and oxaliplatin (FOLFOX) (n=35); FOLFOX and cetuximab (n=6); FU, irinotecan, and leucovorin (LV) (FOLFIRI) (n=12); FOLFIRI and bevacizumab (n=10). 63 patients received 4-6 courses of systematic chemotherapy.

**MRI parameters**

Parameters for IDEAL IQ were as follows: Related parameters were: flip angle=5; echo time=1.3, 3.3, 5.3, 7.3, 9.3, and 11.3 milliseconds; repetition time (TR) =13.7ms; bandwidth=61.25 kHz; slice thickness (SS) =10 mm; matrix size = 256x128; and number of slices=20. Single breath-hold collection was controlled within 18s and then the fat fraction maps could be automatically obtained. And

Parameters for IVIM were as follows: with ten b values of 0, 20, 50, 80, 100, 150, 200, 400, 800, 1000 sec/mm2 respectively. Respiratory triggering was performed using an air-filled pressure sensor and respiration waveform was detected and monitored real-time. Other scanning parameters included Field of view (FOV) =375 x 320 mm; SS =7mm; interslice gap =7 mm, TR =1500ms, echo time (TE) =65ms, NEX =2; and number of slices =6. And all patients were supine and were instructed to breathe smoothly.

**Results**

**Description of Population**

Of 63 patients, 41 subjects with histopathological samples entered into the following study flow. 22 men (54%), 19 women (46%), with a mean age of 54.4 years (range 32–71 years). Mean body mass index was 27.8 kg/m^2^ (range, 18.4–33.1 kg/m^2^). Blood specimens and MRI imaging were performed within 3 days before NC and from liver resection (or RFA), respectively. NC regimens of these 41 patients were as follows: FOLFOX (n=24), of 24 patients, FOLFOX and cetuximab (n=4); FOLFIRI (n=17), of 17 patients, FOLFIRI and bevacizumab (n=4).The time duration of NC ranged from 42 to 130 days (median, 86 days).

**Statistical description of effective biomarkers diagnosing NC-induced liver injury**

And the three effective biomarkers including 25OH-VitD3, *D*, FF values were derived by NC-induced liver injury. 25OH-VitD3 had an AUC of 0.868 (95%CI: 0.736, 0.999). A diagnostic threshold of 8.5 (ng/ml) provided 100% sensitivity (cross-validated), 61.8% specifcity (cross-validated), 100% PPV (cross-validated), and 35% NPV (cross-validated). *D* had an AUC of 0.824 (95%CI: 0.663, 0.984). A diagnostic threshold of 0.93(x10^-3^mm^2^/s) provided 71.4% sensitivity (cross-validated), 82.4% specifcity (cross-validated), 93.33% PPV (cross-validated), and 45.45% NPV (cross-validated). FF values had an AUC of 0.962 (95%CI: 0.899, 1.000). A diagnostic threshold of 7.06% provided 94.1% sensitivity (cross-validated), 85.7% specifcity (cross-validated), 96.97% PPV (cross-validated), and 75% NPV (cross-validated).

**Table S1 Statistical description of effective biomarkers diagnosing NC-induced liver injury**

| **Effective biomarker** | | **N** | **Mean** | **Std. Deviation** | **Std. Error** | **95% Cl** | | **Mini**  **mum** | **Maxi**  **mum** |
| --- | --- | --- | --- | --- | --- | --- | --- | --- | --- |
|  |  |  |  |  |  | **Lower Bound** | **Upper Bound** |  |  |
| **25OH-VitD3** | **0** | 7 | 10.971 | 2.172 | .821 | 8.962 | 12.981 | 8.4 | 14.5 |
|  | **1** | 23 | 8.600 | 1.702 | .355 | 7.864 | 9.336 | 5.3 | 10.7 |
|  | **2** | 11 | 5.236 | 1.413 | .426 | 4.287 | 6.186 | 3.4 | 7.3 |
|  | **Total** | 41 | 8.102 | 2.577 | .402 | 7.289 | 8.916 | 3.4 | 14.5 |
| **FF** | **0** | 7 | 5.602 | 1.882 | .711 | 3.861 | 7.343 | 4.120 | 9.340 |
|  | **1** | 23 | 11.251 | 3.556 | .741 | 9.713 | 12.789 | 5.470 | 18.310 |
|  | **2** | 11 | 18.187 | 3.050 | .919 | 16.137 | 20.236 | 14.100 | 24.840 |
|  | **Total** | 41 | 12.147 | 5.272 | .823 | 10.483 | 13.812 | 4.120 | 24.840 |
| ***D*** | **0** | 7 | .938 | .127 | .0481 | .820 | 1.056 | .705 | 1.073 |
|  | **1** | 23 | .766 | .157 | .0328 | .698 | .834 | .429 | .986 |
|  | **2** | 11 | .674 | .241 | .0727 | .5128 | .836 | .421 | 1.017 |
|  | **Total** | 41 | .771 | .195 | .030 | .709 | .833 | .421 | 1.073 |
